# Association of estimated muscle mass and its changes with all-cause mortality: a Chinese population-based cohort study

**DOI:** 10.1186/s12877-026-07008-6

**Published:** 2026-01-22

**Authors:** Rongxiu Ding, Pan Ding, Chao Lin

**Affiliations:** 1 School and Hospital of Stomatology, Wenzhou Medical University, Wenzhou, Zhejiang P.R. China; 2 School of Mental Health, Wenzhou Medical University, Zhejiang, China; 3 Department of Clinical Laboratory, Key Laboratory of Clinical Laboratory Diagnosis and Translational Research of Zhejiang Province, the First Affiliated Hospital of Wenzhou Medical University, Wenzhou, Zhejiang China

**Keywords:** Skeletal muscle index, All-cause mortality, CLHLS, Chinese elderly

## Abstract

**Background:**

Previous studies have shown an association between low muscle mass (LMM) and all-cause mortality, but sex-specific patterns remain controversial. Moreover, the association between muscle mass changes and all-cause mortality has not been well established. This study aimed to examine the associations of muscle mass and its changes with all-cause mortality.

**Methods:**

Data were derived from the Chinese Longitudinal Healthy Longevity Survey (CLHLS) conducted between 2011 and 2014, with follow-up until 2019. A total of 7,051 participants were included (53.8% female; mean age 83.4 ± 11.0 years). Appendicular skeletal muscle mass (ASM) was estimated using anthropometry-based prediction equations incorporating age, sex, calf circumference, height, and weight. Skeletal muscle index (SMI) was calculated as ASM divided by height squared and categorized into quartiles. Muscle mass changes between 2011 and 2014 were classified into four groups according to sex-specific SMI cut-off values for LMM: persistent LMM, LMM to normal, normal to LMM, and persistent normal. Demographic characteristics, lifestyle factors, and health status indicators were adjusted as covariates. Cox proportional hazards models were used to examine the associations of estimated SMI and its changes with all-cause mortality.

**Results:**

During 33,001 person-years of follow-up, 2,417 deaths occurred, including 1,054 (32.4%) among males and 1,363 (35.9%) among females. The association between SMI and all-cause mortality differed by sex, showing an L-shaped pattern in males and an inverse linear pattern in females. After sex stratification, low SMI was associated with a higher risk of all-cause mortality in females (*P* for interaction < 0.001). Compared with the highest SMI quartile (Q4), the hazard ratios (*HRs*; 95% confidence intervals [*CIs*]) for all-cause mortality in the lowest quartile (Q1) were 1.42 (1.13, 1.79) in males and 1.85 (1.45, 2.36) in females. Additionally, participants with persistent LMM had an increased risk of all-cause mortality compared with those with persistently normal muscle mass in both males (*HR* 1.42, 95%*CI*: 1.17, 1.74) and females (*HR* 1.31, 95%*CI*: 1.04, 1.65).

**Conclusions:**

Low muscle mass may serve as a sensitive indicator of all-cause mortality, and maintaining normal muscle mass may confer longevity-related health benefits.

**Supplementary Information:**

The online version contains supplementary material available at 10.1186/s12877-026-07008-6.

## Introduction

Skeletal muscle is an indispensable component for maintaining bodily function, movement, and balance [1]. As one of the largest metabolic organs in the human body, skeletal muscle is responsible for approximately 80% of postprandial glucose uptake [2]. Appendicular skeletal muscle mass (ASM) plays a crucial role in overall health, yet its changes are often overlooked. Previous studies have demonstrated that a decline in ASM is a hallmark of human aging [3]. Evidence suggested that ASM reaches its peak in early to middle adulthood, begins to decline gradually around the age of 45, and accelerates after 60 years of age, typically decreasing by 3% to 8% per decade [4]. Muscle mass loss can lead to physical dysfunction, reduced quality of life [1], and increased risk of falls [5], frailty [6], and death [7].

Despite accumulating evidence indicating that low muscle mass (LMM) is a predictor of all-cause mortality [8, 9], controversy remains regarding whether the health effects of ASM differ by sex. Some studies have reported an inverse linear association between skeletal muscle mass and all-cause mortality in both males and females [10]. In contrast, other studies have found that skeletal muscle index (SMI) is associated with all-cause mortality in males, showing either an L-shaped [11] or inverse linear association [12], while no significant association was observed in females. Moreover, several studies have even suggested that muscle mass is not associated with all-cause mortality [13]. These inconsistencies may be partly attributable to differences in study populations, limited sample sizes, short follow-up durations, and heterogeneous definitions of muscle mass. In particular, evidence regarding the association between SMI and all-cause mortality among older adults in China remains scarce, highlighting the need for further investigation.

In addition to static measurements, dynamic changes in muscle mass over time may provide greater clinical and public health relevance. Previous studies have shown that continuous muscle mass decline is associated with adverse health outcomes, including lipid metabolism disorders [14, 15] and physical dysfunction [16]. Although the absolute change in SMI calculated from the difference between two measurement results can intuitively reflect the increase or decrease in muscle mass, this method may not fully capture clinically meaningful transition processes: transitioning from LMM to normal muscle mass, remaining in the LMM state, or maintaining normal muscle mass over the long term. Therefore, assessing categorical changes in muscle mass may offer a more clinically meaningful understanding of the association between muscle mass changes and all-cause mortality.

Using a nationally representative sample of Chinese adults aged 65 years and older from the Chinese Longitudinal Healthy Longevity Survey (CLHLS), and after comprehensive adjustment for potential confounders including demographic characteristics, lifestyle factors, and health status, this study aimed to: (1) compare sex differences in the risk of all-cause mortality; (2) examine sex-specific trends in the association between SMI and all-cause mortality; and (3) assess the association between muscle mass changes and all-cause mortality.

## Methods

### Study design and participants

Data were obtained from the CLHLS. Briefly, the CLHLS was initiated in 1998 as an open, community-based cohort study [17]. It employed a targeted, disproportionate sampling design, with follow-up surveys conducted at intervals of 2–4 years (2000, 2002, 2005, 2008, 2011, 2014, and 2018) [18]. The survey collected information on general demographic characteristics, lifestyle factors, and health status through face-to-face interviews conducted by trained public health physicians or community workers [17, 19]. Participants aged 65 years or older were recruited from 22 provinces, municipalities, and autonomous regions across China. During each follow-up wave, approximately 13.8% of participants were lost to follow-up and were replaced by newly recruited individuals [19].

Because calf circumference was first measured in the 2011 wave, participants recruited in 2011 and newly enrolled in 2014 were eligible for inclusion in the present study. The main analysis was designed to examine the association between SMI and all-cause mortality. Of the 10,896 eligible participants, 1,000 were excluded due to missing follow-up information or absence of death records, 2,702 were excluded because of missing anthropometric measurements (waist circumference, calf circumference, height, or weight) or the presence of outliers, and 143 were excluded due to missing data on most covariates. Consequently, 7,051 participants were included in the main analyses.

The secondary analysis aimed to evaluate the association between muscle mass changes and all-cause mortality. This analysis was restricted to participants who completed both the 2011 and 2014 survey waves. Among 6,066 eligible participants, 1,345 individuals with missing follow-up information or dates of death, 387 individuals with missing anthropometric measurements or the presence of outliers, and 17 individuals with missing data for most covariates were excluded. As a result, 4,317 participants were included in the secondary analysis (*Figure* S1).

The CLHLS was approved by the research ethics committee of Peking University (IRB00001052-13074). Written informed consent was obtained from all participants. For illiterate participants, informed consent was provided by their legal guardians or next of kin. All study procedures were conducted in accordance with relevant ethical guidelines and regulations.

### Assessment of Appendicular Skeletal Muscle Mass (ASM), Skeletal Muscle Index (SMI), and Low Muscle Mass (LMM)

The ASM was calculated using a prediction model provided by previous studies (including age, sex, calf circumference, height, and weight) [20]. The prediction formula for the ASM (kg) was: [-0.028*age (year)] + [-3.973*sex (male = 1, female = 2)] + (0.097*weight) + [0.148*height (cm)] + 0.147*calf circumference (cm) − 8.734 (*R*^*2*^ = 0.861, standard error of estimate = 1.53 kg) [21]. The estimated Skeletal Muscle Index (SMI, kg/m^2^) was obtained by further adjusting the square of height (m^2^) based on ASM [21]. Following the recommendations of the Asian Working Group for Sarcopenia (AWGS), LMM was defined as an SMI of less than 7.0 kg/m² in males and 5.4 kg/m² in females [22]. In the main analyses, To facilitate a comparison of the dose relationship between different SMI levels and all-cause mortality, we categorized SMI into 4 groups based on median and quartiles [23] [7.3 (6.9, 7.6) kg/m² for males; 5.2 (4.7, 5.8) kg/m² for females].

In the secondary analyses, muscle mass changes (2011 to 2014 wave) was also categorized into 4 groups: persistent LMM (LMM to LMM), LMM to normal, normal to LMM, and persistent normal (normal to normal), respectively. Previous studies have shown a high correlation (*r* = 0.94) between the measurement of muscle mass using dual-energy X-ray absorptiometry (DXA) and the assessment of muscle mass using variables such as calf circumference [24]. Furthermore, the use of calf circumference to assess muscle mass in community populations can reduce the harms of X-rays, and the Asian Working Group for Sarcopenia has used calf circumference as a case-finding tool for skeletal sarcopenia in primary health care and community health promotion [22].

### Assessment of all-cause mortality

The outcome of this study was all-cause mortality, with follow-up extending until 31 July 2019. Information on survival status and dates of death was obtained through structured interviews with family members, reports from community councils, and verification using official death records. Person-years of follow-up were calculated from the date of completion of the baseline questionnaire in the 2011 (or 2014) survey wave to the date of death, loss to follow-up, or the end of the follow-up period, whichever occurred first [18].

### Assessment of covariates

The covariates considered in this study comprised potential confounders related to demographic characteristics, lifestyle factors, and health status. Demographic characteristics included age (continuous), residence (city, town, rural), education (illiteracy, primary school, middle school or above), widow status (yes, no), economic status (rich, general, poor), and occupation prior to age 60 (technical or service personnel, farming, other). Lifestyle factors encompassed drinking (yes, no), smoking (yes, no), outdoor activity (almost every day, sometimes, never), sleep quality (good, fair, poor), and dietary quality (continuous). Health status indicators included waist circumference (continuous), body mass index (continuous), self-rated health (good, fair, poor), cognitive function (normal, impairment), depression (yes, no), anxiety (yes, no), chronic disease (yes, no), cancer (yes, no), daily activities (normal, impairment), physical functions (continuous), hypertension (yes, no).

Dietary quality was assessed using a 10-item food frequency questionnaire covering fruits, vegetables, fish, red meat, soy and soy products, eggs, sugar, tea, pickles, and garlic. Dietary quality ranged from 10 to 30, with higher scores indicating healthier dietary patterns [19]. Body mass index (BMI) was calculated as weight (kg) divided by height squared (m²) [25]. Cognitive function was evaluated using the 25-item Chinese version of the Mini-Mental State Examination (CMMSE), which assesses orientation and naming, attention, calculation and figure reproduction, language and memory, as well as comprehension and coordination [26]. Given the established influence of educational attainment on cognitive performance, cognitive impairment was defined using education-specific cut-off values: CMMSE scores < 18 for illiterate participants, < 21 for those with primary education, and < 25 for those with middle school education or above; participants with scores above these thresholds were considered cognitively normal [27]. Chronic diseases were defined based on self-reported physician diagnoses of common conditions with high prevalence among older adults in China, including diabetes, asthma, bronchitis, pneumonia, emphysema, heart disease, stroke, or cerebrovascular disease. Participants reporting one or more of these conditions were classified as having a chronic disease [28]. The presence of cancer was also determined by self-report [29]. Daily activities were assessed using the Katz Index of Independence in Activities of Daily Living (ADL), as described in previous studies [29, 30]. Impairment in daily activities was defined as requiring assistance with more than one of the following six activities: dressing, toileting, bathing, continence, indoor transfers, and eating. Physical functions were further evaluated using the 8-item Instrumental Activities of Daily Living (IADL) scale [29], which includes the ability to visit neighbors independently, shop, cook, wash clothes, walk 2 consecutive miles, lift 5 kg, squat and stand up three times consecutively, and use public transportation independently. Higher total IADL scores (range: 0–24) indicated poorer physical functioning.

### Statistical analyses

In main analyses, unordered categorical variables were compared using the chi-square test, whereas ordinal variables and non-normally distributed continuous variables were analyzed using the Kruskal-Wallis test. Restricted cubic spline (RCS) models were applied to examine the dose-response associations between estimated SMI and all-cause mortality. Kaplan-Meier survival curves were used to estimate cumulative mortality across SMI groups, stratified by sex. Cox proportional hazards models were employed to assess the associations of sex and SMI with all-cause mortality, with results reported as hazard ratios (*HRs*) and 95% confidence intervals (*CIs*). Model I was adjusted for demographic characteristics. Model II was further adjusted for lifestyle factors and health status. The proportional hazards assumption was assessed using Schoenfeld residuals, and no significant violations were detected. Multicollinearity among covariates was assessed using variance inflation factors (VIFs). For variables with multiple degrees of freedom, generalized variance inflation factors were adjusted according to their degrees of freedom. All resulting values were below the commonly accepted threshold of 5, indicating that no significant multicollinearity was present among the covariates (*Table* S1). *P* for trend was obtained by modeling the SMI as a continuous data.

To examine the consistency of the associations between SMI and all-cause mortality, stratified analyses were performed according to age, smoking, drinking, sleep quality, outdoor activity, and dietary quality. *P* for interaction was obtained by comparing the difference between models with and without the interaction term. Several sensitivity analyses were conducted to further validate the robustness of the findings. First, to minimize confounding by pre-existing diseases, analyses were restricted to participants without adverse health conditions, defined as having good self-rated health, normal cognitive function, no anxiety or depression, no chronic disease, no cancer, no hypertension, and normal daily activities. Second, to reduce potential reverse causality, participants who died within the first two years of follow-up were excluded to account for a possible lag effect. Third, inverse probability weighting was applied to balance baseline characteristics and enhance comparability across SMI groups.

In secondary analyses, muscle mass changes between the 2011 and 2014 survey waves were classified into four categories. Kaplan-Meier survival curves were used to estimate cumulative mortality across these groups, and Cox proportional hazards models were applied to evaluate the associations between dynamic muscle mass changes and the risk of all-cause mortality.

All data were analyzed using R software version 4.3.1 and EmpowerStats version 4.1 (https://www.empowerstats.net). Statistical significance was defined as a two-sided *P*-value < 0.05.

## Results

In the main analysis, a total of 7,051 Chinese older adults were included (53.8% female; mean age, 83.4 years). During 33,001 person-years of follow-up, 2,417 deaths were recorded, including 1,054 among males and 1,363 among females. The median survival time was 5.2 years for males and 4.6 years for females.

### Descriptive characteristics of participants

In the main analysis, more than half of the participants resided in rural areas, were illiterate, reported a general economic status, had engaged in farming work, were non-smokers and non-drinkers, and reported good sleep quality. Compared with male participants, females were older and had higher proportions of illiteracy, widowhood, farming, non-smoking, non-drinking, no outdoor activity, poor sleep quality, poor self-rated health, poor dietary quality, cognitive impairment, depression, anxiety, impaired activities of daily living, poor physical function, hypertension, and LMM (all *P* < 0.05; Table 1).

**Table 1:** Basic characteristics of participants

Characteristics	Total (*n* = 7,051)	Male (*n* = 3,257)	Female (*n* = 3,794)	*P*-value
Age, years, mean ± SD	83.4 ± 11.0	80.7 ± 9.9	85.6 ± 11.3	**< 0.001**
Residence, *n* (%)				0.337
City	934 (13.2)	431 (13.2)	503 (13.3)	
Town	2,028 (28.8)	964 (29.6)	1,064 (28.0)	
Rural	4,089 (58.0)	1,862 (57.2)	2,227 (58.7)	
Education, *n* (%)				**< 0.001**
Illiterate	4,023 (57.1)	1,061 (32.6)	2,962 (78.1)	
Primary school	1,670 (23.7)	1,153 (35.4)	517 (13.6)	
Middle school or above	1,358 (19.3)	1,043 (32.0)	315 (8.3)	
Widowed, *n* (%)	3,171 (45.0)	1,146 (35.2)	2,734 (72.1)	**< 0.001**
Economic status, *n* (%)				0.106
Rich	1,232 (17.5)	605 (18.6)	627 (16.5)	
General	4,848 (68.8)	2,232 (68.5)	2,616 (69.0)	
Poor	971 (13.8)	420 (12.9)	551 (14.5)	
Occupation prior to age 60, *n* (%)				**< 0.001**
Technical or service personnel	1,113 (15.8)	754 (23.2)	359 (9.5)	
Farming	5,335 (75.7)	2,292 (70.4)	3,043 (80.2)	
Other	603 (8.6)	211 (6.5)	392 (10.3)	
Drinking, *n* (%)	1,343 (19.0)	1,015 (31.2)	328 (8.6)	**< 0.001**
Smoking, *n* (%)	1,370 (19.4)	1,153 (35.4)	217 (5.7)	**< 0.001**
Outdoor activity, *n* (%)				**< 0.001**
Almost everyday	3,330 (47.2)	1,738 (53.4)	1,592 (42.0)	
Sometimes	1,317 (18.7)	586 (18.0)	731 (19.3)	
Never	2,404 (34.1)	933 (28.6)	1,471 (38.8)	
Sleep quality, *n* (%)				**< 0.001**
Good	4,464 (63.3)	2,256 (69.3)	2,208 (58.2)	
Fair	1,778 (25.2)	722 (22.2)	1,056 (27.8)	
Poor	809 (11.5)	279 (8.6)	530 (14.0)	
Dietary quality, mean ± SD	22.5 ± 3.2	23.0 ± 3.2	22.1 ± 3.1	**< 0.001**
Waist circumference, cm, mean ± SD	81.9 ± 11.0	83.0 ± 10.1	80.9 ± 11.5	**< 0.001**
Body mass index, kg/m^2^, mean ± SD	21.5 ± 3.80	21.8 ± 3.42	21.3 ± 4.08	**< 0.001**
Self-rated health, *n* (%)				**< 0.001**
Good	3,256 (46.2)	1,627 (50.0)	1,629 (42.9)	
Fair	2,831 (40.2)	1,279 (39.3)	1,552 (40.9)	
Poor	964 (13.7)	351 (10.8)	613 (16.2)	
Cognitive impairment, *n* (%)	1,306 (18.5)	398 (12.2)	908 (23.9)	**< 0.001**
Depression, *n* (%)	816 (11.6)	325 (10.0)	491 (12.9)	**< 0.001**
Anxiety, *n* (%)	1,928 (27.3)	714 (21.9)	1,214 (32.0)	**< 0.001**
Chronic disease, *n* (%)	2,322 (32.9)	1,100 (33.8)	1,222 (32.2)	0.171
Cancer, *n* (%)	344 (4.9)	166 (5.1)	178 (4.7)	0.949
Impaired daily activities, *n* (%)	1,281 (18.2)	448 (13.8)	833 (22.0)	**< 0.001**
Physical functions, mean ± SD	12.1 ± 5.3	10.6 ± 4.4	13.3 ± 5.7	**< 0.001**
Hypertension, *n* (%)	2,573 (36.5)	1,125 (34.5)	1,448 (38.2)	**0.002**
SMI, kg/m^2^, mean ± SD	6.2 ± 1.2	7.3 ± 0.5	5.3 ± 0.8	**< 0.001**
LMM, *n* (%)	3,138 (44.5)	920 (28.2)	2,218 (58.5)	**< 0.001**

*SD *Standard Deviation, *SMI* Skeletal Muscle Index,* LMM *Low Muscle Mass

Unordered categorical data were tested using the Chi-square test, and ordinal and nonnormal continuous data were tested using the Kruskal-Wallis test

The bold values indicated statistical significance *P* < 0.05

In the secondary analysis, participants with persistent LMM were older and had higher proportions of females, rural residence, illiteracy, widowhood, poor economic status, farming work, smoking, never outdoor activity, poor sleep quality, poor dietary quality, poor self-rated health, cognitive impairment, and poor physical functioning (all *P* < 0.05; *Table* S2).

### Association of sex and estimated SMI with risk of all-cause mortality

**Fig. 1:**
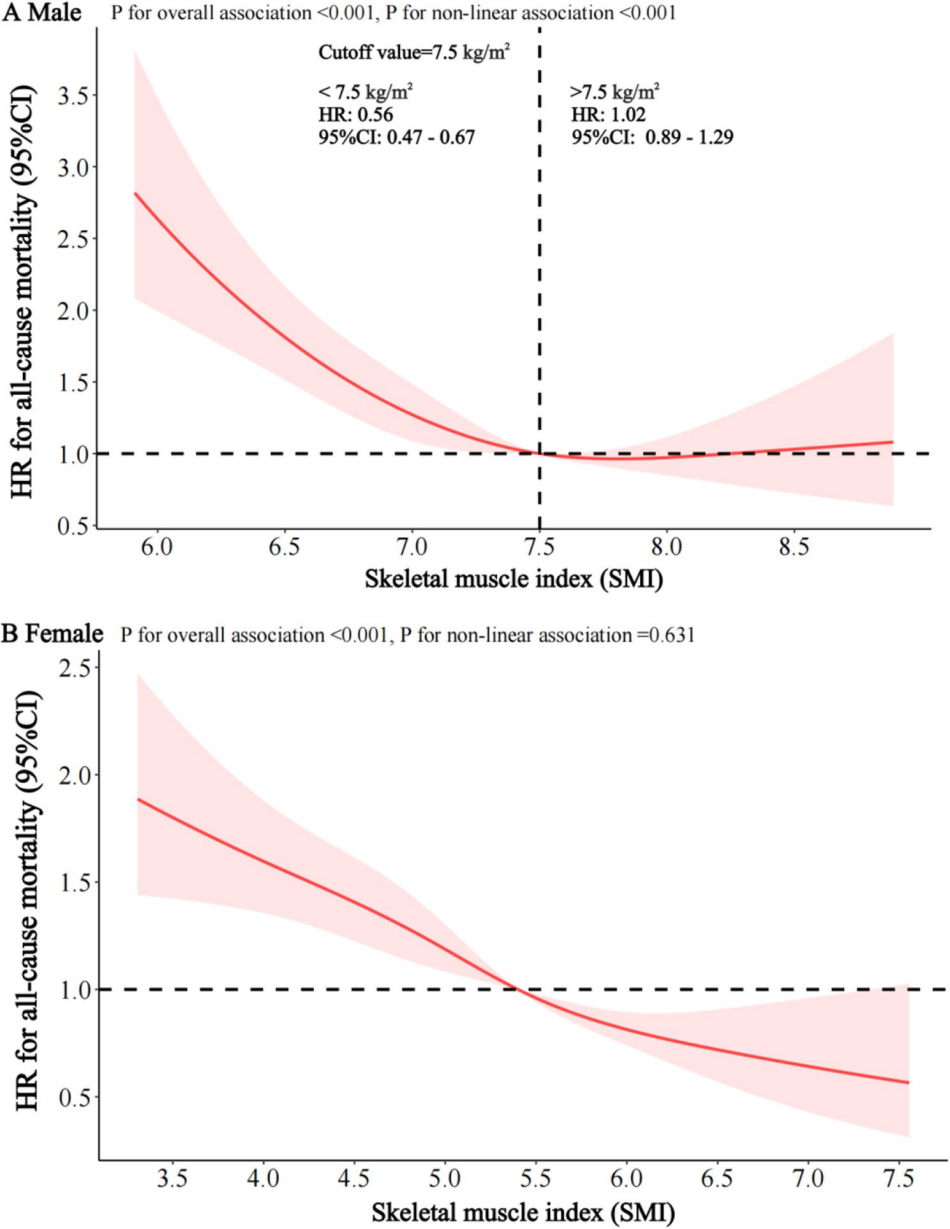
Restricted cubic spline for the association of SMI and all-cause mortality in male and female. Note: (**A**) association of SMI with all-cause mortality in male, (**B**) association of SMI with all-cause mortality in female. The reference value for the male population was 7.5 kg/m2 (based on the threshold effect) and for the female population was 5.4 kg/m2 (based on AWGS recommendations). HR, hazard ratio; CI, confidence interval; SMI, Skeletal Muscle Index; AWGS, the Asian Working Group for Sarcopenia. Spline curves were adjusted for age (continuous), residence (city, town, rural), education (illiteracy, primary school, middle school or above), widow status (yes, no), economic status (rich, general, poor), occupation prior to age 60 (technical or service personnel, farming, other), drinking (yes, no), smoking (yes, no), outdoor activity (almost every day, sometimes, never), sleep quality (good, fair, poor), dietary quality (continuous), waist circumference (continuous), body mass index (continuous), self-rated health (good, fair, poor), cognitive function (normal, impairment), depression (yes, no), anxiety (yes, no), chronic disease (yes, no), cancer (yes, no), daily activities (normal, impairment), physical functions (continuous), hypertension (yes, no)

Restricted cubic spline analyses demonstrated that the associations between estimated SMI and all-cause mortality differed by sex. Among male participants, the association exhibited an L-shaped pattern, whereby increases in SMI were associated with a marked reduction in mortality risk up to a threshold of 7.5 kg/m², beyond which further increases conferred no additional survival benefit (*P* for overall < 0.001, *P* for nonlinear < 0.001). In contrast, among female participants, an inverse linear association was observed, indicating that each incremental increase in SMI was associated with a sustained and proportional reduction in all-cause mortality (*P* for overall < 0.001, *P* for nonlinear = 0.631) (Fig. 1).

As shown in Table 2, after sequential adjustment for demographic characteristics, lifestyle factors, and health status indicators (Model II), males had a higher overall risk of all-cause mortality than females (*HR* 1.76, 95%*CI*: 1.59, 1.94). Sex-stratified analyses further revealed a stronger association between low SMI and all-cause mortality in females (*P* for interaction < 0.001). In Model II, compared with the highest SMI quartile (Q4), the hazard ratios for all-cause mortality in males were 1.42 (1.13, 1.79) for Q1, 1.24 (1.01, 1.54) for Q2, and 1.16 (0.93, 1.44) for Q3, respectively (*P* for trend < 0.001). Correspondingly, in females, the *HRs* (95%*CIs*) were 1.85 (1.45, 2.36) for Q1, 1.54 (1.23, 1.94) for Q2, and 1.39 (1.12, 1.73) for Q3, respectively, compared with Q4 (*P* for trend < 0.001). Consistent with these findings, Kaplan-Meier survival curves stratified by SMI quartiles showed significantly shorter survival times among participants with lower SMI in both males and females (log-rank *P* < 0.05; *Figure* S2).

**Table 2:** Association between SMI and all-cause mortality in male and female

Variables	Cases/person, years	HRs (95%CI)			*P* for interaction
		Crude model	Model I	Model II	
Sex					
Male	1,054/15,466	**1.31 (1.21**,** 1.43)**	**1.51 (1.38**,** 1.66)**	**1.76 (1.59**,** 1.94)**	
Female	1,363/17,535	1.00	1.00	1.00	
SMI (Male)					**< 0.001**
Q1	393/3,536	**3.38 (2.80**,** 4.08)**	**1.46 (1.20**,** 1.78)**	**1.42 (1.13**,** 1.79)**	
Q2	290/3,743	**2.28 (1.87**,** 2.78)**	**1.30 (1.06**,** 1.60)**	**1.24 (1.01**,** 1.54)**	
Q3	221/3,997	**1.58 (1.28**,** 1.94)**	1.16 (0.94, 1.43)	1.16 (0.93, 1.44)	
Q4	150/4,190	1.00	1.00	1.00	
*P* for trend		< 0.001	< 0.001	< 0.001	
SMI (Female)					
Q1	588/3,441	**7.66 (6.34**,** 9.25)**	**1.98 (1.59**,** 2.45)**	**1.85 (1.45**,** 2.36)**	
Q2	388/4,251	**3.74 (3.07**,** 4.56)**	**1.51 (1.22**,** 1.85)**	**1.54 (1.23**,** 1.94)**	
Q3	253/4,764	**2.07 (1.68**,** 2.56)**	**1.32 (1.06**,** 1.63)**	**1.39 (1.12**,** 1.73)**	
Q4	134/5,079	1.00	1.00	1.00	
*P* for trend		< 0.001	< 0.001	< 0.001	

*HR *hazard ratio, *CI *confidence interval,* SMI *Skeletal Muscle Index

Q1 ≤ 6.9 kg/m², Q2 ≤ 7.3 kg/m², Q3 ≤ 7.6 kg/m², and Q4 > 7.6 kg/m² for male; Q1 ≤ 4.7 kg/m², Q2 ≤ 5.2 kg/m², Q3 ≤ 5.8 kg/m², and Q4 > 5.8 kg/m² for female

Model I was adjusted for age (continuous), residence (city, town, rural), education (illiteracy, primary school, middle school or above), widow status (yes, no), economic status (rich, general, poor), occupation prior to age 60 (technical or service personnel, farming, other). Model II model was further adjusted for drinking (yes, no), smoking (yes, no), outdoor activity (almost every day, sometimes, never), sleep quality (good, fair, poor), dietary quality (continuous), waist circumference (continuous), body mass index (continuous), self-rated health (good, fair, poor), cognitive function (normal, impairment), depression (yes, no), anxiety (yes, no), chronic disease (yes, no), cancer (yes, no), daily activities (normal, impairment), physical functions (continuous), hypertension (yes, no)

The bold values indicated statistical significance *P* < 0.05

Additional analyses examining LMM yielded results consistent with the primary SMI-based findings. Participants with LMM had a significantly higher risk of all-cause mortality in both males and females (*Table* S3).

### Stratification and sensitivity analysis

After stratifying by age, drinking, smoking, sleep quality, outdoor activity, and dietary quality, we found that the *HRs* > 1 for the association between low SMI and the risk of all-cause mortality in both male and female populations (all *P* for interaction > 0.05; Table 3).

**Table 3:** Association between SMI and all-cause mortality in male and female, stratification analyses

Subgroups	*n*	Cases/person, years	SMI, HRs (95%CI)				*P* for interaction
			Q1	Q2	Q3	Q4	
Male:							
Age, years							0.181
≤ 80	1,670	279/8,840	**1.63 (1.13**,** 2.35)**	1.23 (0.80, 1.89)	1.21 (0.86, 1.71)	1.00	
> 80	1,587	775/6,626	**1.84 (1.39**,** 2.46)**	**1.34 (1.01**,** 1.78)**	1.27 (0.95, 1.69)	1.00	
Drinking							0.123
No	2,242	781/10,426	**1.50 (1.14**,** 1.96)**	**1.32 (1.03**,** 1.69)**	1.26 (0.97, 1.64)	1.00	
Yes	1,015	273/5,040	1.39 (0.87, 2.23)	1.21 (0.79, 1.87)	1.06 (0.68, 1.63)	1.00	
Smoking							0.128
No	2,104	714/9,814	**1.51 (1.13**,** 2.02)**	**1.42 (1.08**,** 1.86)**	1.26 (0.96, 1.65)	1.00	
Yes	1,153	340/5,652	**1.73 (1.15**,** 2.61)**	1.45 (0.92, 2.28)	1.18 (0.81, 1.71)	1.00	
Sleep quality							0.554
Good	2,256	685/10,722	**1.50 (1.12**,** 2.00)**	1.27 (0.97, 1.65)	1.21 (0.92, 1.60)	1.00	
Fair	722	272/3,339	**1.57 (1.01**,** 2.49)**	1.53 (0.95, 2.45)	1.14 (0.73, 1.77)	1.00	
Poor	279	97/1,405	**1.48 (1.04**,** 2.55)**	1.11 (0.73, 2.21)	1.04 (0.80, 1.83)	1.00	
Outdoor activity							0.345
Almostly everyday	1,738	456/8,799	**1.56 (1.21**,** 2.64)**	**1.42 (1.02**,** 1.98)**	**1.46 (1.06**,** 2.00)**	1.00	
Sometimes	586	189/2,799	**1.40 (1.00**,** 2.23)**	1.23 (0.99, 2.14)	1.06 (0.60, 1.83)	1.00	
Never	933	409/3,968	**1.48 (1.02**,** 2.17)**	1.26 (0.89, 1.83)	1.02 (0.71, 1.46)	1.00	
Dietary quality							0.368
≤ 23	1,706	609/7,958	**1.49 (1.09**,** 2.04)**	**1.39 (1.03**,** 1.89)**	1.27 (0.95, 1.71)	1.00	
> 23	1,551	445/7,508	**1.41 (1.02**,** 2.12)**	1.25 (0.97, 1.82)	1.18 (0.85, 1.64)	1.00	
Female:							
Age, years							0.448
≤ 80	1,382	150/7,687	**2.12 (1.04**,** 4.37)**	**1.85 (1.10**,** 3.10)**	1.45 (0.94, 2.23)	1.00	
> 80	2,412	1,213/9,848	**2.27 (1.75**,** 2.93)**	**1.58 (1.23**,** 2.04)**	1.28 (0.99, 1.67)	1.00	
Drinking							0.790
No	3,466	1,256/15,962	**2.03 (1.58**,** 2.63)**	**1.60 (1.26**,** 2.03)**	**1.39 (1.11**,** 1.75)**	1.00	
Yes	328	107/1,573	**1.86 (1.12**,** 2.93)**	**1.42 (1.03**,** 2.51)**	1.24 (0.84, 2.02)	1.00	
Smoking							0.089
No	3,577	1,292/16,545	**2.01 (1.56**,** 2.59)**	**1.59 (1.25**,** 2.01)**	**1.39 (1.11**,** 1.74)**	1.00	
Yes	217	71/990	1.58 (0.45, 3.57)	1.32 (0.52, 3.35)	1.11 (0.70, 1.95)	1.00	
Sleep quality							0.071
Good	2,208	819/10,046	**2.09 (1.53**,** 2.86)**	**1.72 (1.29**,** 2.30)**	1.32 (0.99, 1.75)	1.00	
Fair	1,056	372/4,801	**2.04 (1.26**,** 3.29)**	1.55 (0.99, 2.42)	**1.24 (0.78**,** 1.97)**	1.00	
Poor	530	172/2,688	**1.75 (1.01**,** 2.66)**	1.40 (0.97, 2.25)	1.40 (0.95, 210)	1.00	
Outdoor activity							0.276
Almostly everyday	1,592	410/7,956	**1.90 (1.22**,** 2.97)**	**1.73 (1.16**,** 2.57)**	**1.68 (1.16**,** 2.44)**	1.00	
Sometimes	731	263/3,439	**1.59 (1.01**,** 2.60)**	1.41 (0.90, 2.23)	1.00 (0.72, 1.64)	1.00	
Never	1,471	690/6,140	**2.07 (1.50**,** 2.86)**	**1.46 (1.06**,** 2.00)**	1.38 (0.99, 1.92)	1.00	
Dietary quality							0.083
≤ 22	2,026	788/9,256	**1.57 (1.14**,** 2.15)**	**1.30 (1.00**,** 1.76)**	1.21 (0.90, 1.61)	1.00	
> 22	1,768	575/8,279	**2.03 (1.60**,** 3.10)**	**1.69 (1.16**,** 2.43)**	**1.46 (1.04**,** 2.05)**	1.00	

*HR *hazard ratio, *CI *confidence interval, *SMI *Skeletal Muscle Index

Q1 ≤ 6.9 kg/m², Q2 ≤ 7.3 kg/m², Q3 ≤ 7.6 kg/m², and Q4 > 7.6 kg/m² for male; Q1 ≤ 4.7 kg/m², Q2 ≤ 5.2 kg/m², Q3 ≤ 5.8 kg/m², and Q4 > 5.8 kg/m² for female

Adjusted for age (continuous), residence (city, town, rural), education (illiteracy, primary school, middle school or above), widow status (yes, no), economic status (rich, general, poor), occupation prior to age 60 (technical or service personnel, farming, other), drinking (yes, no), smoking (yes, no), outdoor activity (almost every day, sometimes, never), sleep quality (good, fair, poor), dietary quality (continuous), waist circumference (continuous), body mass index (continuous), self-rated health (good, fair, poor), cognitive function (normal, impairment), depression (yes, no), anxiety (yes, no), chronic disease (yes, no), cancer (yes, no), daily activities (normal, impairment), physical functions (continuous), hypertension (yes, no)

The bold values indicated statistical significance *P* < 0.05

In further sensitivity analyses, we first restricted the population to individuals in good health. Secondly, we excluded participants who had died within two years. Finally, we applied inverse probability weighting to account for confounding factors. Ultimately, we found that the association between low SMI and all-cause mortality remained robust (*Table* S4).

### Association between muscle mass changes and all-cause mortality

In a secondary analysis, survival curves showed the fastest rate of decline in the persistent LMM population among the muscle mass change groups (*Figure* S3). After stratifying by sex, in further multivariable-adjusted models, we found that persistent LMM was associated with a high risk of all-cause mortality in both the male (*HR* 1.42, 95%*CI*: 1.17, 1.74) and female (*HR* 1.31, 95%*CI*: 1.04, 1.65) populations compared with participants with persistent normal muscle mass (Table 4).

**Table 4:** Association between muscle mass changes and all-cause mortality

Muscle mass changes (2011 to 2014)	*n*	Cases/person, years	^a^ HRs (95%CI)
Male:			
Persistent LMM	926	391/5,294	**1.42 (1.17**,** 1.74)**
LMM to Normal	254	84/1,500	1.13 (0.86, 1.48)
Normal to LMM	238	73/1,413	1.00 (0.73, 1.35)
Persistent Normal	652	152/3,994	1.00
Female:			
Persistent LMM	1,208	504/6,951	**1.31 (1.04**,** 1.65)**
LMM to Normal	239	84/1,453	1.12 (0.89, 1.38)
Normal to LMM	301	81/1,838	1.08 (0.81, 1.44)
Persistent Normal	499	95/3,168	1.00

*HR *hazard ratio, *CI *confidence interval, *LMM *low muscle mass

^a^ Adjusted for sex (male, female), age (continuous), residence (city, town, rural), education (illiteracy, primary school, middle school or above), widow status (yes, no), economic status (rich, general, poor), occupation prior to age 60 (technical or service personnel, farming, other), drinking (yes, no), smoking (yes, no), outdoor activity (almost every day, sometimes, never), sleep quality (good, fair, poor), dietary quality (continuous), waist circumference (continuous), body mass index (continuous), self-rated health (good, fair, poor), cognitive function (normal, impairment), depression (yes, no), anxiety (yes, no), chronic disease (yes, no), cancer (yes, no), daily activities (normal, impairment), physical functions (continuous), and hypertension (yes, no)

The bold values indicated statistical significance *P* < 0.05

## Discussion

### Main findings

In this 2011 to 2018 wave of the Chinese Community Cohort Study, our finding was that older male adults had a 76% higher risk of mortality than females. After sex stratification, we found that the risk of all-cause mortality was higher with low SMI in the females. After adjusting for general demographic characteristics, lifestyle, and health status confounders, the dose-response trends for estimated SMI and the risk of all-cause mortality differed between male and female populations. Males exhibited an L-shaped pattern (threshold of 7.5 kg/m^2^), while females showed an inverse linear pattern, with a 42% and 85% increase in the risk of mortality, respectively, for those with low SMI. Furthermore, compared with participants with persistently normal muscle mass, participants with persistently LMM had a 42% increased risk of all-cause mortality in males and a 31% increased risk in females. To the best of our knowledge, this is the first study to investigate the association between muscle mass index and its variation with the risk of all-cause mortality in a community-based elderly population in China.

### Comparison with other studies

In our study, the overall prevalence of LMM was 44.5%, with a markedly higher prevalence among females (58.5%) than males (28.2%). Several recent studies have reported comparable overall prevalence estimates. For example, a Korean study applying the AWGS criteria reported an overall LMM prevalence of 41.0% (40.3% in males and 41.3% in females) [31], while a Pakistani study using X-ray-based assessments reported an overall prevalence of 47.2% (60.0% in males and 36.4% in females) [32]. The higher prevalence of LMM among older Chinese women observed in our study may be attributable to advanced age, a higher proportion of illiteracy, poorer dietary quality, and generally poorer health status. In addition, differences in SMI calculation methods and sample sizes may partly explain the observed variability across studies. By contrast, a UK-based prospective study of 4,102 community-dwelling older adults (mean age 69.7 years) reported a substantially lower LMM prevalence of 13.9% over a 10-year follow-up period [33]. Variations in cohort design, population ethnicity, age distribution, SMI assessment methods, and diagnostic thresholds likely contributed to this discrepancy.

Sex differences in all-cause mortality have been extensively examined, with substantial evidence indicating longer survival among females than males [34, 35]. Recent cohort studies from China [36] and Canada [37] reported that males have a 33%-42% higher risk of all-cause mortality compared with females, which is consistent with our findings. However, studies from the United States [38] and China [39] have reported no significant sex differences in all-cause mortality among individuals with cardiovascular disease. Given that cardiovascular disease is a major determinant of mortality, its presence may attenuate or obscure the independent effect of sex on survival outcomes.

Accumulating evidence has demonstrated that low SMI is associated with multiple adverse health outcomes, including systemic inflammation [40], metabolic dysfunction [41], osteoporosis [42], and fracture risk [43]. A recent meta-analysis encompassing 16 studies and 81,358 participants confirmed that low SMI is significantly associated with increased all-cause mortality [9], in line with our results. Similarly, Japanese cohort studies have identified low SMI as an independent predictor of all-cause mortality [10]. In contrast, a Korean study suggested that physical function indicators, such as gait speed and sit-to-stand performance, may be stronger predictors of mortality than muscle mass or strength alone [44]. While separate assessments of muscle mass, strength, and physical function provide complementary information, muscle mass decline remains a fundamental contributor to functional deterioration in older adults.

Notably, the association patterns between SMI and all-cause mortality appear to differ by sex. A Japanese study reported an L-shaped association between SMI and mortality in males, whereas a similar but non-significant trend was observed in females [11]. Seino et al. [10] further reported an inverse dose-response relationship between SMI and mortality in both sexes, findings that are broadly consistent with our observations. Differences in age structure, ethnicity, geographic region, and dietary patterns may underlie the heterogeneity in association patterns across studies. Therefore, future large-scale prospective studies with broader age ranges and regional representation are warranted to further clarify sex-specific relationships between SMI and all-cause mortality.

A large prospective study from the Kailuan Cohort in China (*n* = 101,510) reported that [45] individuals with persistently high muscle mass had a 47.4% lower risk of all-cause mortality compared to those with persistently low muscle mass (*P* < 0.001). These findings are consistent with our results, which also suggest that persistently low muscle mass is associated with an increased risk of mortality. In contrast, a Korean study among cancer patients (*n* = 4,056) demonstrated that [46] simultaneous increases in both body weight and muscle mass were associated with a lower risk of mortality (*HR* 0.68; *P* = 0.001) compared to individuals with stable weight and muscle mass, whereas weight loss accompanied by muscle mass reduction was linked to a higher risk of mortality (*HR* 1.73; *P* < 0.001). However, no such association was observed in our study. Several factors may explain these discrepancies. Firstly, the Korean study focused on cancer patients, a population more prone to disease-related cachexia and rapid muscle loss, whereas our study population comprised community-dwelling older adults, whose muscle mass changes may be more gradual. Secondly, the Korean study quantified muscle mass using computed tomography imaging, whereas our study estimated muscle mass via a multivariate equation based on anthropometric measurements. These methodological differences may have influenced the sensitivity for detecting short-term muscle mass changes and their association with mortality.

### Potential mechanisms

Low SMI may increase the risk of all-cause mortality through multiple potential pathways, including direct effects on physical function and overall health status, as well as interactions with chronic diseases. Firstly, with advancing age, hormonal alterations (e.g., testosterone, estrogen, and insulin-like growth factor-1) disrupt the balance between muscle protein synthesis and degradation [47, 48], thereby promoting the development of sarcopenia. Secondly, skeletal muscle is a major metabolic organ that plays a critical role in glucose and lipid metabolism [49]. Loss of muscle mass may lead to metabolic dysfunction [50], increasing the risk of diabetes, metabolic syndrome, and cardiovascular diseases. Thirdly, sarcopenia is associated with chronic low-grade inflammation, where elevated levels of inflammatory markers [50, 51] (e.g., C-reactive protein and interleukin-6) may accelerate muscle loss and are also linked to a higher risk of various chronic diseases (such as cardiovascular diseases [52] and cancer [45]). Finally, LMM is frequently accompanied by malnutrition [40], which may reduce physical activity capacity [53], increase the risk of falls [1] and fractures [54], and contribute to the coexistence of multiple chronic diseases [45] (e.g., cardiovascular diseases, diabetes, cancer), thereby ultimately elevating mortality risk.

### Strengths and limitations

Our study has several strengths, including a prospective cohort design based on a Chinese community, a large-scale participant population, comprehensive adjustment for covariates, and repeated measurement data.

However, several limitations should be acknowledged. First, SMI was estimated using a predictive model derived from previous studies. Although this approach has been widely applied and validated in large epidemiological investigations [55, 56], direct measurements provide more precise assessments of muscle mass. Therefore, caution is warranted when interpreting differences between SMI estimated using multivariable models and those obtained from direct measurement techniques. Second, although we adjusted for a wide range of potential confounders, including physical functioning, multiple chronic conditions, and mental health status, the CLHLS did not collect information on several important sarcopenia-related indicators, such as grip strength, muscle weakness, fatigue, gait speed, or cause-specific mortality. These limitations restricted our ability to comprehensively evaluate sarcopenia and to explore specific pathways linking muscle mass to mortality risk. Future studies incorporating more detailed functional and clinical measures are needed. Third, lifestyle variables such as smoking and alcohol consumption were assessed in the CLHLS using binary self-reported measures (yes/no), without detailed information on duration, intensity, or cumulative exposure. This may have led to residual confounding and limited our ability to assess potential dose–response relationships between these behaviors, muscle mass, and mortality outcomes. Finally, although a two-year lag analysis was conducted to reduce potential reverse causality, the overall follow-up duration remained relatively limited. Consequently, causal inferences regarding the long-term health effects of muscle mass and its dynamic changes should be interpreted with caution. Longer-term prospective studies are warranted to further validate these associations.

## Conclusions

Based on this study, we observed a higher risk of all-cause mortality among males. Low SMI was associated with an increased risk of all-cause mortality in both males and females. Moreover, the association between estimated SMI and all-cause mortality differed by sex, displaying an L-shaped pattern in males and an inverse linear association in females. Persistent LMM was also associated with an elevated risk of all-cause mortality. Collectively, these findings indicate that LMM may serve as a sensitive indicator of mortality risk. Early identification of community-dwelling individuals with persistent LMM may hold important implications for improving health outcomes among older populations.

## Supplementary Information

Supplementary Material 1.

## Data Availability

All data used in this study were accessed from the publicly available Chinese Longitudinal Healthy Longevity Survey: https://opendata.pku.edu.cn/dataverse/CHADS.

## Abbreviations

LMM: Low Muscle Mass
CLHLS: Chinese Longitudinal Healthy Longevity Survey
ASM: Appendicular Skeletal Muscle mass
SMI: Skeletal Muscle Index
AWGS: The Asian Working Group for Sarcopenia
DXA: Dual-energy X-ray Absorptiometry
BMI: Body Mass Index
CMMSE: Chinese version of the Mini-Mental State Examination
ADL: Independence in Activities of Daily Living
IADL: Instrumental Activities of Daily Living
RCS: Restricted Cubic Spline
HRs: Hazard Ratios
CIs: Confidence Intervals
VIFs: Variance Inflation Factors
