# Supplementary material for: Association of estimated muscle mass and its changes with all-cause mortality: a Chinese population-based cohort study
Source: BMC Geriatr. 2026 Jan 22;26:393. doi: 10.1186/s12877-026-07008-6 (PMC13014735; doi:10.1186/s12877-026-07008-6)
Supplement: Supplementary file 1 — Supplementary Material 1. [file 12877_2026_7008_MOESM1_ESM.docx]

**Figure S1: Flow chart of the study population, a prospective cohort**

Participants surveyed in 2011 or 2014, *n*=10,896

Participants were included in the main study, *n*=7,051

Exclude those who lost to follow-up, *n*=1,000

Exclude those who missed muscle mass variables in the 2011 or 2014 surveys, *n*=2,702

Participants included in the follow-up, *n*=9,896

Participants both surveyed in 2011 and 2014, *n*=6,066

Exclude those who lost to follow-up, *n*=1,345

Participants included in the follow-up, *n*=4,721

Participants were included in the second study, *n*=4,317

Exclude those who missed muscle mass variables in the 2011 or 2014 surveys, *n*=387

**Main analysis**

**Secondary analysis**

Exclude those who were missing most variables information, *n*=143

Exclude who were missing most variables information, *n*=17

**Table S1: Multicollinearity Assessment (GVIFs) in the Multivariable Cox Model**

| **Variables** | **GVIF** | **Df** | **GVIF^(1/(2*Df))** |
| --- | --- | --- | --- |
| Skeletal Muscle Index | 1.720167 | 3 | 1.094616 |
| Occupation | 1.475640 | 2 | 1.102161 |
| Education | 1.333324 | 2 | 1.074568 |
| Economic status | 1.384220 | 2 | 1.084679 |
| Residence | 1.341728 | 2 | 1.076257 |
| Widow status | 1.280372 | 1 | 1.131535 |
| Age | 1.752207 | 1 | 1.323710 |
| Drinking | 1.101686 | 1 | 1.049612 |
| Smoking | 1.125432 | 1 | 1.060864 |
| Sleep quality | 1.202932 | 2 | 1.047274 |
| Outdoor activity | 1.275010 | 2 | 1.062621 |
| Dietary quality | 1.208283 | 1 | 1.099219 |
| Self-rated health | 1.397857 | 2 | 1.087341 |
| Depression | 1.147672 | 1 | 1.071294 |
| Anxiety | 1.216764 | 1 | 1.103070 |
| Cognitive function | 1.497688 | 1 | 1.223801 |
| Daily activities | 1.547133 | 1 | 1.243838 |
| Physical functions | 2.360049 | 1 | 1.536245 |
| Chronic disease | 1.269645 | 1 | 1.126785 |
| Hypertension | 1.218271 | 1 | 1.103753 |
| Cancer | 1.224728 | 2 | 1.051986 |
| Waist circumference | 1.598350 | 1 | 1.264259 |
| Body mass index | 2.054209 | 1 | 1.433251 |

|  |
| --- |

**Table S2: Baseline characteristics of participants in 2011-2014 waves**

| **Characteristics** | **Total**  **(n=4317)** | **Normal→Normal**  **(n=1151)** | **Normal→LMM**  **(n=539)** | **LMM→Normal**  **(n=493)** | **LMM→LMM**  **(n=2134)** | **P-value** |
| --- | --- | --- | --- | --- | --- | --- |
| **Age, years, mean±SD** | 81.4±10.3 | 76.1±8.61 | 81.2±9.20 | 80.7±10.0 | 84.4±10.4 | **<0.001** |
| **Male, *n* (%)** | 2070 (48.0) | 652 (56.6) | 238 (44.2) | 254 (51.5) | 926 (43.4) | **<0.001** |
| **Residence, *n* (%)** |  |  |  |  |  | **<0.001** |
| City | 538 (12.5) | 221 (19.2) | 71 (13.2) | 46 (9.3) | 200 (9.4) |  |
| Town | 1334 (30.9) | 339 (29.5) | 168 (31.2) | 159 (32.3) | 668 (31.3) |  |
| Rural | 2445 (56.6) | 591 (51.3) | 300 (55.7) | 288 (58.4) | 1266 (59.3) |  |
| **Education, *n* (%)** |  |  |  |  |  | **<0.001** |
| Illiterate | 2315 (53.6) | 474 (41.2) | 295 (54.7) | 250 (50.7) | 1296 (60.7) |  |
| Primary school | 1143 (26.5) | 313 (27.2) | 149 (27.6) | 144 (29.2) | 537 (25.2) |  |
| Middle school or above | 859 (19.9) | 364 (31.6) | 95 (17.6) | 99 (20.1) | 301 (14.1) |  |
| **Widowed, *n* (%)** | 2171 (50.3) | 418 (36.3) | 265 (49.2) | 229 (46.5) | 1259 (59.0) | **<0.001** |
| **Economic status, *n* (%)** |  |  |  |  |  | **0.002** |
| Rich | 764 (17.7) | 238 (20.7) | 86 (16.0) | 84 (17.0) | 356 (16.7) |  |
| General | 2914 (67.5) | 785 (68.2) | 372 (69.0) | 320 (64.9) | 1437 (67.3) |  |
| Poor | 639 (14.8) | 128 (11.1) | 81 (15.0) | 89 (18.1) | 341 (16.0) |  |
| **Occupation prior to age 60, *n* (%)** |  |  |  |  |  | **<0.001** |
| Technical or service personnel | 666 (15.4) | 272 (23.6) | 89 (16.5) | 63 (12.8) | 242 (11.3) |  |
| Farming | 3284 (76.1) | 774 (67.2) | 409 (75.9) | 386 (78.3) | 1715 (80.4) |  |
| Other | 367 (8.5) | 105 (9.1) | 41 (7.6) | 44 (8.9) | 177 (8.3) |  |
| **Drinking, *n* (%)** | 874 (20.2) | 261 (22.7) | 89 (16.5) | 107 (21.7) | 417 (19.5) | **0.038** |
| **Smoking, *n* (%)** | 898 (20.8) | 241 (20.9) | 86 (16.0) | 115 (23.3) | 456 (21.4) | **0.040** |
| **Outdoor activity, *n* (%)** |  |  |  |  |  | **<0.001** |
| Almost everyday | 2178 (50.5) | 652 (56.6) | 283 (52.5) | 250 (50.7) | 993 (46.5) |  |
| Sometimes | 805 (18.6) | 187 (16.2) | 107 (19.9) | 95 (19.3) | 416 (19.5) |  |
| Never | 1334 (30.9) | 312 (27.1) | 149 (27.6) | 148 (30.0) | 725 (34.0) |  |
| **Sleep quality, *n* (%)** |  |  |  |  |  | **<0.001** |
| Good | 2688 (62.3) | 767 (66.6) | 341 (63.3) | 320 (64.9) | 1260 (59.0) |  |
| Fair | 1081 (25.0) | 263 (22.8) | 143 (26.5) | 108 (21.9) | 567 (26.6) |  |
| Poor | 548 (12.7) | 121 (10.5) | 55 (10.2) | 65 (13.2) | 307 (14.4) |  |
| **Diet score, mean±SD** | 22.6±3.23 | 23.4±3.13 | 22.7±3.17 | 22.2±3.41 | 22.2±3.19 | **<0.001** |
| **Waist circumference, cm, mean±SD** | 82.3±11.0 | 88.0±11.1 | 80.7±10.7 | 84.2±10.9 | 79.3±9.70 | **<0.001** |
| **Body mass index, kg/m^2^, mean±SD** | 21.7±3.80 | 24.7±3.44 | 23.3±3.80 | 21.1±2.99 | 19.8±2.83 | **<0.001** |
| **Self-rated health, *n* (%)** |  |  |  |  |  | **<0.001** |
| Good | 2004 (46.4) | 592 (51.4) | 242 (44.9) | 248 (50.3) | 922 (43.2) |  |
| Fair | 1700 (39.4) | 426 (37.0) | 221 (41.0) | 178 (36.1) | 875 (41.0) |  |
| Poor | 613 (14.2) | 133 (11.6) | 76 (14.1) | 67 (13.6) | 337 (15.8) |  |
| **Cognitive impairment, *n* (%)** | 589 (13.6) | 77 (6.7) | 68 (12.6) | 61 (12.4) | 383 (17.9) | **<0.001** |
| **Depression, *n* (%)** | 570 (13.2) | 148 (12.9) | 61 (11.3) | 70 (14.2) | 291 (13.6) | 0.632 |
| **Anxiety, *n* (%)** | 1023 (23.7) | 240 (20.9) | 131 (24.3) | 130 (26.4) | 522 (24.5) | 0.095 |
| **Chronic disease, *n* (%)** | 1452 (33.6) | 468 (40.7) | 191 (35.4) | 156 (31.6) | 637 (29.9) | **<0.001** |
| **Cancer, *n* (%)** | 253 (5.9) | 88 (7.7) | 29 (5.4) | 27 (5.5) | 109 (5.1) | 0.055 |
| **Impaired daily activities, *n* (%)** | 603 (14.0) | 135 (11.7) | 70 (13.0) | 70 (14.2) | 328 (15.4) | 0.068 |
| **Physical functions, mean±SD** | 11.1±4.58 | 9.83±3.76 | 10.9±4.41 | 10.9±4.53 | 11.8±4.88 | **<0.001** |
| **Hypertension, *n* (%)** | 1648 (38.2) | 550 (47.8) | 210 (39.0) | 191 (38.7) | 697 (32.7) | **<0.001** |

**Note:** SD, Standard Deviation; LMM, Low Muscle Mass. Unordered categorical data were tested using the Chi-square test, and ordinal and nonnormal continuous data were tested using the Kruskal-Wallis test. The bold values indicated statistical significance P<0.05.

**Table S3: Association between low muscle mass (LMM) and all-cause mortality**

| **Variables** | Cases/person, years | ***HRs* (95%*CI*)** | | |
| --- | --- | --- | --- | --- |
|  |  | Crude model | Model I | Model II |
| Male | 1,054/15,466 |  |  |  |
| Normal | 627/11,422 | 1.00 | 1.00 | 1.00 |
| LMM | 427/4,044 | **2.03 (1.79, 2.29)** | **1.20 (1.06, 1.37)** | **1.21 (1.05, 1.39)** |
| Female | 1,363/17,535 |  |  |  |
| Normal | 293/8,336 | 1.00 | 1.00 | 1.00 |
| LMM | 1,070/9,199 | **3.63 (3.19, 4.14)** | **1.43 (1.24, 1.65)** | **1.36 (1.16, 1.59)** |

Note: HR, hazard ratio; CI, confidence interval; LMM, low muscle mass.

Model I was adjusted for age (continuous), residence (city, town, rural), education (illiteracy, primary school, middle school or above), widow status (yes, no), economic status (rich, general, poor), occupation prior to age 60 (technical or service personnel, farming, other). Model II model was further adjusted for drinking (yes, no), smoking (yes, no), outdoor activity (almost every day, sometimes, never), sleep quality (good, fair, poor), diet score (continuous), waist circumference (continuous), body mass index (continuous), self-rated health (good, fair, poor), cognitive function (normal, impairment), depression (yes, no), anxiety (yes, no), chronic disease (yes, no), cancer (yes, no), daily activities (normal, impairment), physical functions (continuous), hypertension (yes, no).

The bold values indicated statistical significance *P*<0.05.

| **Outcomes** | ***n*** | **Cases/person, years** | **SMI, *HRs* (95%*CI*)** | | | |
| --- | --- | --- | --- | --- | --- | --- |
|  |  |  | **Q1** | **Q2** | **Q3** | **Q4** |
| **Male:** | | | | | | |
| **1. ^a^ Limiting population to:** | | | | | | |
| Good self, rated health | 1,627 | 435/7,868 | **1.52 (1.06, 2.18)** | **1.53 (1.09, 2.15)** | 1.24 (0.89, 1.72) | 1.00 |
| Normal cognitive function | 2,859 | 815/13,948 | **1.52 (1.18, 1.95)** | 1.23 (0.97, 1.56) | 1.15 (0.91, 1.46) | 1.00 |
| No anxiety | 2,543 | 782/12,333 | **1.41 (1.12, 1.78)** | **1.28 (1.02, 1.63)** | 1.16 (0.92, 1.47) | 1.00 |
| No depression | 2,932 | 950/13,797 | **1.46 (1.14, 1.88)** | **1.31 (1.04, 1.65)** | 1.17 (0.93, 1.47) | 1.00 |
| No chronic disease | 2,157 | 676/10,340 | **1.35 (1.01, 1.81)** | 1.23 (0.93, 1.63) | 1.07 (0.81, 1.42) | 1.00 |
| No cancer | 3,074 | 183/14,575 | **1.48 (1.16, 1.89)** | **1.29 (1.03, 1.63)** | 1.17 (0.94, 1.47) | 1.00 |
| No hypertension | 2,132 | 708/9,964 | **1.53 (1.17, 2.00)** | 1.29 (0.98, 1.69) | 1.22 (0.92, 1.62) | 1.00 |
| Normal daily activities | 2,809 | 801/13,701 | **1.42 (1.12, 1.79)** | **1.28 (1.01, 1.63)** | 1.13 (0.89, 1.44) | 1.00 |
| **2. ^a^ Exclusion of people who died within two years:** | | | | | | |
|  | 3,006 | 848/15,158 | **1.72 (1.33, 2.23)** | **1.36 (1.07, 1.74)** | 1.25 (0.99, 1.59) | 1.00 |
| **3. ^b^ Inverse probability weighting:** | | | | | | |
|  | 3,257 | 1,054/15,466 | **1.58 (1.23, 2.02)** | **1.36 (1.06, 1.76)** | 1.21 (0.93, 1.57) | 1.00 |
| **Female:** | | | | | | |
| **1. ^a^ Limiting population to:** | | | | | | |
| Good self, rated health | 1,629 | 537/7,648 | **2.70 (1.80, 4.06)** | **1.94 (1.33, 2.83)** | **1.59 (1.10, 2.30)** | 1.00 |
| Normal cognitive function | 2,886 | 776/14,282 | **1.91 (1.42, 2.58)** | **1.52 (1.15, 2.00)** | **1.36 (1.05, 1.76)** | 1.00 |
| No anxiety | 2,580 | 853/12,276 | **2.02 (1.48, 2.75)** | **1.58 (1.18, 2.12)** | **1.35 (1.02, 1.79)** | 1.00 |
| No depression | 3,303 | 1,223/14,943 | **1.98 (1.53, 2.57)** | **1.57 (1.23, 2.00)** | **1.40 (1.11, 1.77)** | 1.00 |
| No chronic disease | 2,572 | 942/11,753 | **1.77 (1.30, 2.42)** | **1.47 (1.10, 1.97)** | 1.24 (0.93, 1.66) | 1.00 |
| No cancer | 3,594 | 200/16,555 | **1.85 (1.44, 2.38)** | **1.51 (1.20, 1.91)** | **1.35 (1.08, 1.69)** | 1.00 |
| No hypertension | 2,346 | 899/10,609 | **2.04 (1.48, 2.80)** | **1.64 (1.21, 2.23)** | 1.31 (0.97, 1.78) | 1.00 |
| Normal daily activities | 2,961 | 873/14,440 | **1.88 (1.40, 2.53)** | **1.56 (1.19, 2.04)** | **1.36 (1.05, 1.75)** | 1.00 |
| **2. ^a^ Exclusion of people who died within two years** | | | | | | |
|  | 3,392 | 1,016/17,050 | **1.95 (1.49, 2.56)** | **1.60 (1.25, 2.06)** | **1.45 (1.14, 1.83)** | 1.00 |
| **3. ^b^ Inverse probability weighting:** | | | | | | |
|  | 3,794 | 1,363/17,535 | **1.90 (1.49, 2.42)** | **1.52 (1.21, 1.91)** | **1.33 (1.07, 1.66)** | 1.00 |

**Table S4: Sensitivity analyses of the association between SMI and all-cause mortality in male and female**

Note: HR, hazard ratio; CI, confidence interval; SMI, Skeletal Muscle Index.

Q1 ≤6.9 kg/m², Q2 ≤7.3 kg/m², Q3 ≤7.6 kg/m², and Q4 >7.6 kg/m² for male; Q1 ≤4.7 kg/m², Q2 ≤5.2 kg/m², Q3 ≤5.8 kg/m², and Q4 >5.8 kg/m² for female.

^a^ Adjusted for age (continuous), residence (city, town, rural), education (illiteracy, primary school, middle school or above), widow status (yes, no), economic status (rich, general, poor), drinking (yes, no), smoking (yes, no), outdoor activity (almost every day, sometimes, never), sleep quality (good, fair, poor), diet score (continuous), waist circumference (continuous), body mass index (continuous), self-rated health (good, fair, poor), cognitive function (normal, impairment), depression (yes, no), anxiety (yes, no), chronic disease (yes, no), cancer (yes, no), daily activities (normal, impairment), physical functions (continuous), hypertension (yes, no).

^b^ Propensity weight was based on: age (continuous), residence (city, town, rural), education (illiteracy, primary school, middle school or above), widow status (yes, no), economic status (rich, general, poor), drinking (yes, no), smoking (yes, no), outdoor activity (almost every day, sometimes, never), sleep quality (good, fair, poor), diet score (continuous), waist circumference (continuous), body mass index (continuous), self-rated health (good, fair, poor), cognitive function (normal, impairment), depression (yes, no), anxiety (yes, no), chronic disease (yes, no), cancer (yes, no), daily activities (normal, impairment), physical functions (continuous), hypertension (yes, no).

The bold values indicated statistical significance *P*<0.05.

**Figure S2: Kaplan-Meier survival curves for male and female populations with risk of all-cause mortality after interquartile grouping for SMI**


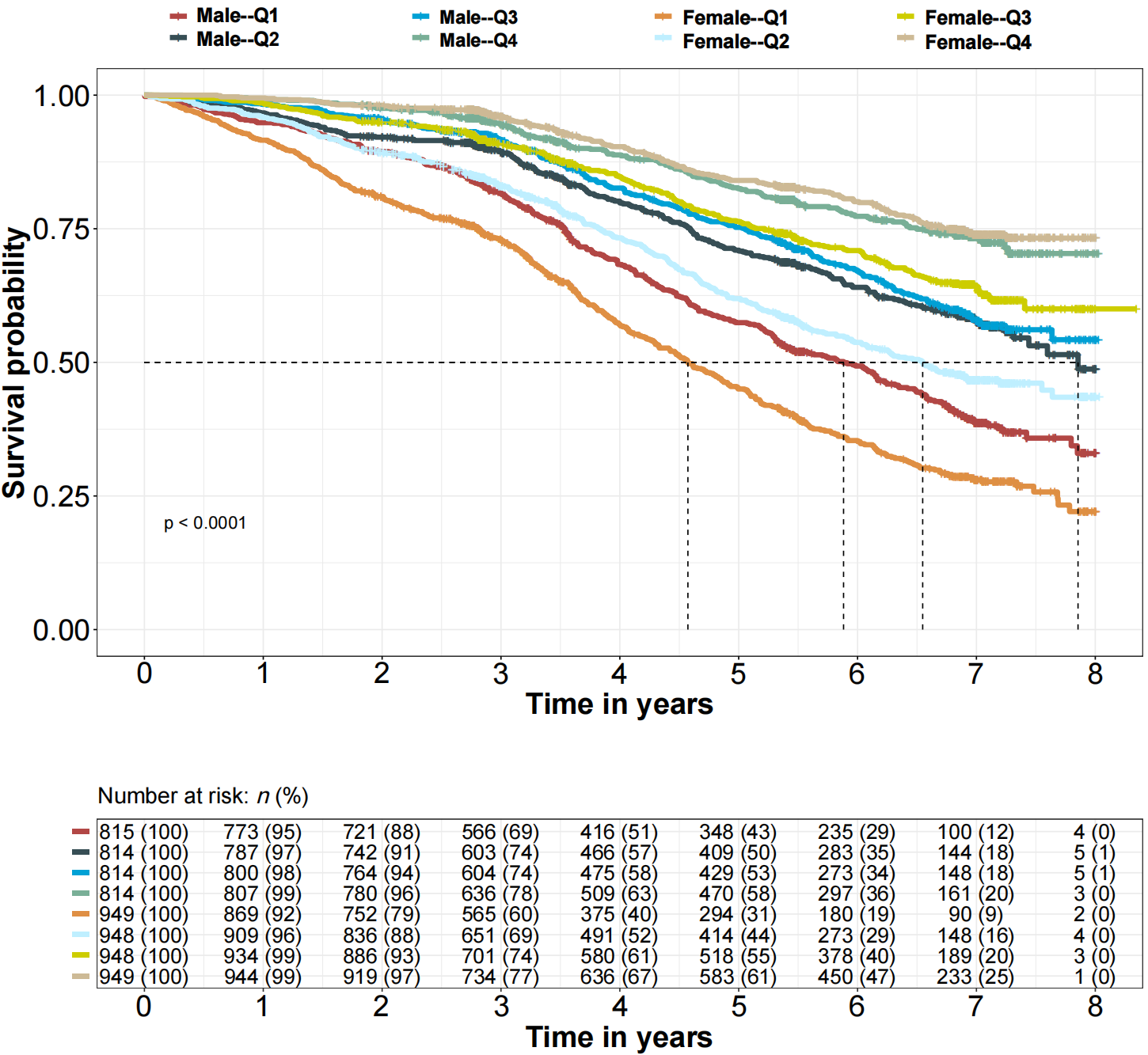


Note: Kaplan-Meier survival curves for male and female populations, stratified by sex and SMI interquartile groups, illustrating all-cause mortality risk over 8 years. Significance (p < 0.0001) indicates differences in survival probabilities across groups.

**Figure S3: Kaplan-Meier survival curves for muscle mass change and all-cause mortality**


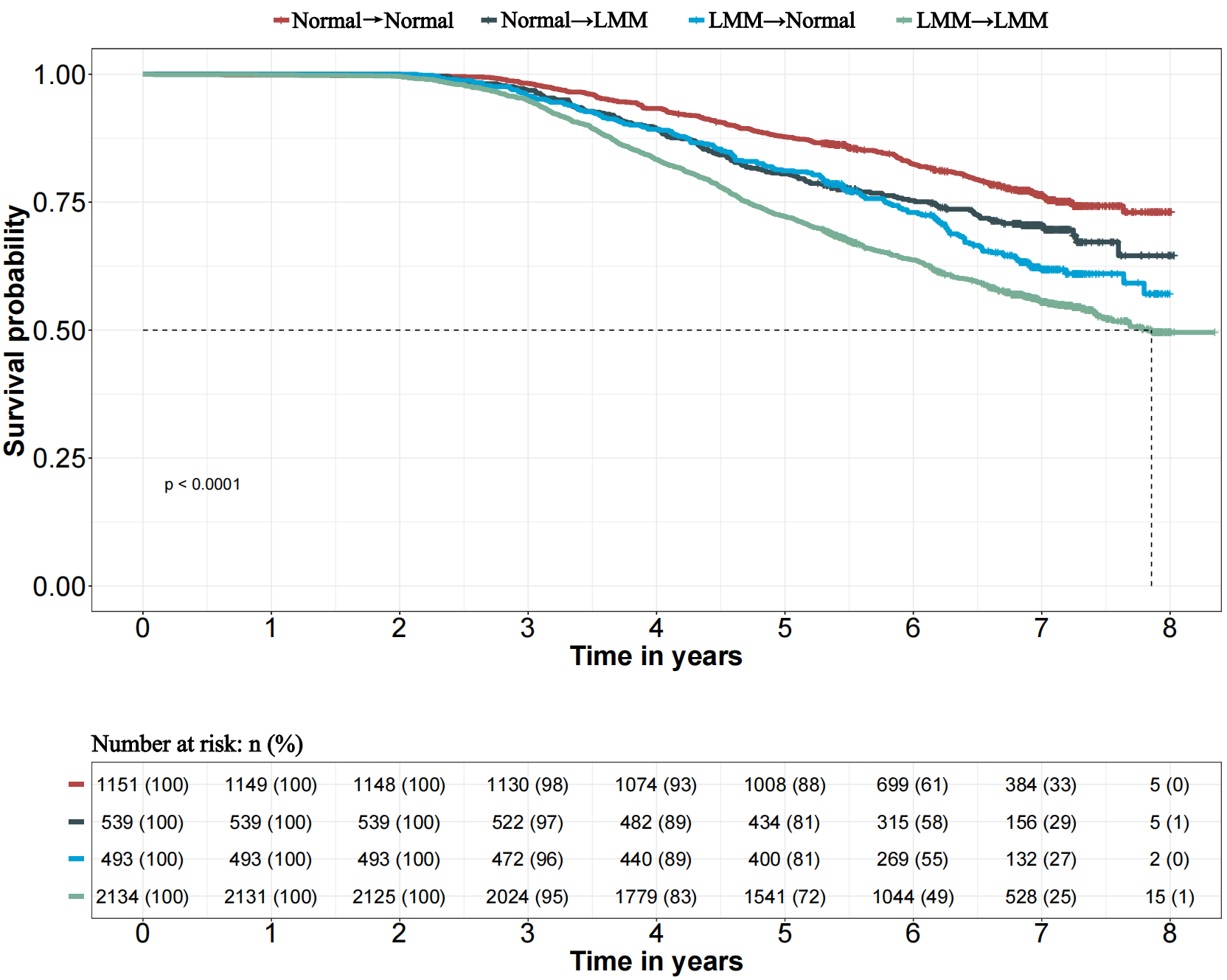


Note: Kaplan-Meier survival curves stratified by muscle mass change patterns (Normal→Normal, Normal→LMM, LMM→Normal, LMM→LMM), demonstrating all-cause mortality risk over 8 years. A significant difference (p < 0.0001) indicates varying survival probabilities across muscle mass change groups.
